# Nanoemulsion-based plant essential oil formulations: in vitro evaluation of pesticidal activity against ectoparasites in poultry

**DOI:** 10.1016/j.psj.2023.103245

**Published:** 2023-10-30

**Authors:** Anuwat Lakyat, Jarongsak Pumnuan, Thanaporn Doungnapa, Sudjai Phutphat, Somsak Kramchote, Kamronwit Thipmanee

**Affiliations:** ⁎ School of Agricultural Technology, King Mongkut's Institute of Technology Ladkrabang, Bangkok 10520, Thailand; † School of Science, King Mongkut's Institute of Technology Ladkrabang, Bangkok 10520, Thailand

**Keywords:** clove, cinnamon, nanoemulsion, ectoparasite, poultry

## Abstract

Ectoparasite infestations significantly impact the health and productivity of poultry. Chemical applications, although common for pest control, lead to pesticide residues and parasite resistance in poultry. Nanoemulsion-based plant essential oil formulations (**NEOFs**) provide a promising alternative for controlling poultry ectoparasites. This study aimed to assess the efficacy of NEOFs from clove, cinnamon, and turmeric essential oils (**EOs**) against ectoparasites, *Menopon gallinae* and *Megninia ginglymura*, under laboratory conditions. The toxicity and repellent properties of the NEOFs were examined, with the major chemical compounds of the EOs analyzed using chromatography mass spectrometer. Results identified eugenol as the dominant component in clove and cinnamon EOs (84.60 and 75.19%, respectively), while turmerone (68.46%) was the major compound in turmeric EO. NEOFs with clove:cinnamon:turmeric ratios of 4:0:0, 2:2:0, and 2:0:2 had particle size of 20.76 nm, 20.66 nm, and 89.56 nm, respectively, while those based on eugenol and turmerone standards had sizes <21.0 nm. In addition, NEOFs at 0.3% concentration with ratios of 4:0:0 and 2:2:0 achieved full control of both ectoparasites. These formulas demonstrated exceptional potency in exterminating ectoparasites, with LC_50_ and LC_90_ at <0.160 and <0.250%, respectively, 6 h after treatments. Furthermore, both NEOFs showed higher repellence responses in *M. gallinae* compared to *M. ginglymura*. The toxicities of these NEOFs were comparably effective against both parasites, showing no significant difference compared with chemical insecticide treatment. Therefore, further research will explore the practicality of using clove and cinnamon-derived NEOFs under farm conditions.

## INTRODUCTION

Poultry farming is vital to Thailand, being a major revenue source within the country's livestock sector. As one of the world's leading producers of agricultural commodities, Thailand's poultry industry is the second largest in Southeast Asia and is expected to expand further (Netherlands Embassy in Bangkok, 2016). Most of poultry population and production are located in central Thailand, with the integrated commercial farms accounting for 80 to 90% of national production (Rushton et al., 2007). Meanwhile, traditional poultry scattered across the country primarily cater to local consumption (Heft-Neal et al., 2009). A key challenge impacting the economic success of poultry farming is ectoparasitic infestation. These external parasites can cause significant health issues for poultry, including reduced growth, lower egg production, and in severe cases, death (McCrea et al., 2005; Onyekachi, 2021). Common poultry ectoparasites include lice, mites, fleas, and ticks, all of which negatively impact reproductive potential, reduced egg production and health of poultry. Their feeding behaviors can cause irritation, restlessness and debility, and severe infestations may result in fatal anemia (Salam et al., 2009; Mirzaei et al., 2016). In Bangkok, Thailand, several reports indicated that chicken lice, *Lipeurus caponis, Menopon gallinae*, and *Goniocotes gallinae*, are the most prevalent poultry ectoparasites, while *Megninia cubitalis* being the predominant species of chicken mite (Lakyat et al., 2022). Traditionally, pesticides such as pyrethroids, organophosphates, carbamates, and macrocyclic lactones have been widely used for ectoparasitic control in poultry. However, these agents have led to resistance among parasites (Coles and Dryden, 2014; Sparagano et al., 2022), and their use has resulted in harmful environmental residues and potential toxicity to humans and animals (Sharma et al., 2019). Despite the widespread use of these pesticides, none provide complete protection against poultry ectoparasites. This necessitates the exploration of alternative, eco-friendly pesticides, potentially derived from medicinal plants with pesticidal properties (Shanta et al., 2008; Pumnuan et al., 2020). Poultry treated with chemical pesticides often produce eggs contaminated with toxicity residues. A study conducted by Alaboudi et al. (2019) revealed that 96% of egg samples from a total of 200 households with laying hens in Jordan, were contaminated with pesticides. Concerningly, 66.5% of these samples showed contamination levels exceeding the maximum residue limit (**MRL**) of less than 0.01 ppm. In particular, cypermethrin, an insecticide, had the highest incidence being present in 52% of egg samples from Rio Grande do Sul in 2015 (Dallegrave et al., 2018). However, such applications provide inadequate protection against poultry ectoparasites. Consequently, research is now focusing on highly effective, human-safe, and environmentally friendly alternative green pesticides with no toxic residues in meat or eggs. These pesticides are derived from plant EOs with pesticidal properties (Pumnuan et al., 2020).

Several papers reported that various plant EOs showed high efficiency in controlling poultry ectoparasites. For instance, EOs of ginger and citronella eliminated the highest effectiveness against lice (*M. gallinae*) and mites (*Ornithonyssus bursa*) (Vigad et al., 2021). Especially, EOs of clove, cinnamon, and turmeric effectively killed lice (*Lipeurus caponis*) completely after 12 h of exposure (Pumnuan et al., 2020). Furthermore, the main chemicals in plant EOs, including botanical pesticides, are recognized for their potential in controlling a wide range of pests (Isman, 2006). Specifically, eugenol, a compound present in clove and cinnamon EOs, has been shown to have potent insecticidal and acaricidal properties (Fichi et al., 2007a,b; Abenaim et al., 2022). For example, eugenol has demonstrated significant ovicidal activities against parasitic mites (*Sarcoptes scabiei*) (Li et al., 2021). Studies have suggested that clove and cinnamon EOs, and their components, could be used to develop fumigants, repellents, and attractants to control poultry red mite, *Dermanyssus gallinae* (Sparagano et al., 2013; Lee et al., 2019).

Therefore, this research aimed to evaluate the effectiveness of EOs obtained from 3 plants; clove, cinnamon, and turmeric, and their major compounds. These would be incorporated into farming formulas, based on nanoemulsion, to control ectoparasites infesting laying hens under laboratory conditions.

## MATERIALS AND METHODS

### Preparation of Plant Essential Oil-Based Nanoemulsions

Essential oils (**EOs**) were obtained from 3 dried medicinal plants: clove buds (*Syzygium aromaticum*), cinnamon leaves (*Cinnamomum zeylanicum*), and turmeric rhizomes (*Curcuma longa*). The selection was based on previous research and academic papers about their efficacy against ectoparasites (Pumnuan et al., 2020). Nanoemulsions of these EOs (**NEOs**) were prepared according to the methodology described by Doungnapa et al. (2021). Specifically, nanoemulsion of clove (**NEO-CL**) and cinnamon (**NEO-CI**) were prepared with EO:Surfactant:Cosurfactant ratios of 2:9:2, while for turmeric (**NEO-TU**), the ratio was 2:6:3. All NEOs, at a 1.0% concentration in water, were then evaluated for particle size and zeta potential.

### Ectoparasite Used

The ectoparasites *Menopon gallinae* (shaft louse) and *Megninia ginglymura* (chicken mite) were collected from the Smart Chicken Farm at the School of Agricultural Technology, King Mongkut's Institute of Technology Ladkrabang (**KMITL**), Thailand. Adult ectoparasites were screened from chicken feathers and skin using an aspirator, and tested in the laboratory within 2 h of collection.

### Chemical Characterization of Plant Essential Oils

The EOs extracted from clove, cinnamon and turmeric, were analyzed using Gas Chromatography Mass Spectrometer (**GC-MS**) (Agilent Technologies Inc., USA). The GC-MS was equipped with an HP5MS capillary column (30 m length × 0.25 mm I.D. × 0.25 µm film thickness). Analysis parameters included direct injection of a 0.4 µl volume, a split mode with a split ratio of 50:1 v/v, and an injection temperature of 250°C. Helium served as a carrier gas with a flow rate of 1 mL/min and an ionization voltage of 70 eV. Mass range detection was set to 50 to 500 *m*/*z*. The oven temperature, started at 50°C, held for 3 min, then increased by 10°C/min until reaching 200°C, after which it was raised by 15°C/min until it reached 260°C. The detector was maintained at 270°C. The results obtained were compared with those in Wiley's library (**Wiley7n**), accepting a quality match of over 85%.

Nanoemulsions of the main compound standard (**NCS**) were prepared using clove, cinnamon and turmeric EOs. Eugenol (**E**) was identified as the primary constituent of clove and cinnamon EOs, while turmerone (**T**) was present in turmeric EO. NCS-E, containing eugenol (Fluka Analytical) was prepared in the same ratio as NEO-CL and NEO-CI. Similarly, NCS-T, consisting of turmerone (MedChemExpress), was prepared in the same ratio as NEO-TU. The particle size of the NCS at 1.0% in water were was measured, along with their polydispersity index (**PDI**) and zeta potential by using a Nano plus Zeta/Nano Particle Analyzer as done in NEO experiments.

### Preparation of Nanoemulsion

Based on a report by Pumnuan et al. (2020), clove EO has demonstrated higher efficacy in killing chicken lice (*Liperus caponis*) compared to cinnamon and turmeric EOs. Consequently, a formula called NEO-CL was prepared as the main component, while NEO-CI and NEO-TU were used as secondary components. These mixtures were referred to as nanoemulsion-based plant essential oil formulations (**NEOFs**). Four different ratios of mixture were prepared as NEO-CL:NEO-CI:NEO-TU, namely 4:0:0, 2:2:0, 2:0:2 and 2:1:1, respectively. Subsequently, the NEOFs at 1.0% in water were analyzed for the particle size and zeta potential.

### Particle Size and Zeta Potential Assessment and Morphological Analysis

The particle size (diameter), PDI, and zeta potential of NEO, NCS, and NEOF at 1% in water were measured using a Nano plus Zeta/Nano Particle Analyzer (Micromeritics Instrument Corporation, Japan) with the manufacturer's software.

The morphological structure of all nanoemulsion-based plant EO formulations was observed using Field Emission Transmission Electron Microscopy (**FE-TEM**) (Thermo Fisher Scientific: Talos F200i, Czech Republic). A drop of the 1% nanoemulsion-based sample in water was placed on a copper grid for 1 min. The sample was then stained with 2% uranyl acetate for 10 min at room temperature. Subsequently, the copper grid with the sample was placed in vacuum chamber to absorb moisture overnight. Finally, the sample was observed under the FE-TEM at an acceleration voltage 200 kV.

### Contact Toxicity Bioassays

The lethal contact residue exposure test was performed according to the method described by Pumnuan et al. (2020). A total of 1 mL of each NEOF at 3.0% was dropped onto a 9 cm diameter filter paper (Whatman; No.1) and placed in a glass petri dish. The dishes were dried at room temperature (25°C ± 3°C) for 5 min, and then 10 adults of each poultry ectoparasites (shaft louse, *M. gallinae* and chicken mites, *M. ginglymura*) were released. The petri dishes were tightly closed and wrapped with parafilm and kept at room temperature. The percentage mortality was measured at 3 and 6 h, and the actual mortality was calculated and compared with the control group (0.25% surfactant in water). The 2 formulas with the highest efficiency in killing ectoparasites were further tested for toxicity at concentrations of 0.15, 0.20, 0.25, 0.30, and 0.35%. The actual mortality rate was checked and compared with that of the control group. The toxicity level was calculated to determine the LC_50_ and LC_90_ values. Data comparisons were also made with the insecticide group (cypermethrin 35% EC, recommendation rate; 0.1%) and NCS. The experimental design was carried out using a completely randomized design (**CRD**), with 3 replicates.

### Repellency Bioassays

NEOFs selected from previous contact residue exposure tests were examined for their repellency and attractant activities against 2 species of ectoparasites: shaft louse and the feather mites. The experimental design was carried out using CRD, by performing at 0.15 and 0.25% NEOFs, with 3 replicates.

For the repellent and attractant tests on the shaft louse, feathers were dipped in each concentration of the test solution for 1 min, dried at room temperature (25°C ± 3°C) for 5 min, and then released with 10 adult shaft lice per feather. The petri dishes were tightly covered, wrapped with parafilm and placed at room temperature. The number of shaft lice on the feathers and on the filter paper (control) was counted at 3 and 6 h, and the repellent and attractant rates were calculated.

The repellent test on chicken mites was performed using a glass test tube (0.5 cm diameter, 8 cm length) with one end attached to a filter paper and moistened with each concentration of each NEOF, while the other end was moistened with the control group (0.25% surfactant in water). The treated papers were moistened for 1 min and dried at room temperature for 5 min, and then 10 adult mites per tube were inserted. The tubes were placed at room temperature, and the number of mites on each side of the tube was evaluated at 3 and 6 h to determine the repellent and attractant rate.

### Data Analysis

The experiment was designed in 3 randomized replicates. The data obtained were statistically analyzed using analysis of variance (**ANOVA**), and the difference between treatments was tested by Duncan's multiple range test (**DMRT**). The actual death rates were calculated via Abbot's formula (Abbott, 1987). The toxicity test results, represented by LC_50_ and LC_90_ values (lethal concentration of NEOFs required to kill 50 and 90% of ectoparasites, respectively) were determined using probit analysis. The data from the repellency were analyzed by the χ^2^ test.

## RESULTS

### Chemical Characterization of Plant Essential Oils

The GC-MS analysis of the EOs obtained from the clove, cinnamon, and turmeric revealed the major compounds present in each oil. Eugenol was found to be the predominant compound in clove and cinnamon EOs, constituting 84.60 and 75.19% of the oils, respectively. Turmerone emerged as a major compound in turmeric EO, comprising 68.46% of the oil. In addition to the major compounds, minor chemical compounds were also detected in the EOs (Table 1).

**Table 1:** The main components in clove, cinnamon, and turmeric essential oils.

Clove		Cinnamon		Turmeric	
Chemical components	%	Chemical components	%	Chemical components	%
Eugenol	84.60	Eugenol	75.19	Turmerone	68.46
Caryophyllene	10.06	Benzyl benzoate	4.20	Cyclohexane	6.37
*Alpha*-Humulene	2.09	Caryophyllene	3.66	Zingiberene	5.27
Caryophyllene oxide	0.69	Linalool L	2.23	*Alpha*-Terpinolene	3.71
*Delta*-Cadinene	0.52	*trans*-Cinnamyl acetate	2.18	Curcumene	3.17
Other compounds	2.04	o-Cymene	1.77	1-Phellandrene	2.45
		Cinnamaldehyde	1.71	Cyclododecene	1.19
		Safrole	1.50	1,8-Cineole	1.76
		*Alpha*-Pinene	1.21	*Trans*-Caryophyllene	1.26
		Copaene	0.91	*Beta*-Cymene	1.00
		*Beta*-Thujene	0.74	*Alpha*-Atlantone	0.62
		*Alpha*-Humulene	0.70	Bisabolone (6S, 7R)	0.56
		Caryophyllene oxide	0.60	Other compounds	4.18
		Anethol	0.52		
		Rubicene	0.51		
		Other compounds	2.37		

### Particle Size and Zeta Potential Assessment and Morphological Analysis

The particle size, PDI, and zeta potential of the nanoemulsions containing NEOs, NCSs, and NEOFs at a concentration of 1.0% in water were determined. The particle size of all NEOs from clove, cinnamon, and turmeric, as well as NCSs derived from eugenol and turmerone chemical standards, ranged from 17.15 to 20.80 nm. The NEOF-1, NEOF-2, and NEOF-3 formulations, which were mixtures of NEO-CL:NEO-CI:NEO-TU with ratios of 4:0:0, 2:2:0, and 2:0:2, respectively, exhibited particle sizes of 20.76 nm, 20.66 nm, and 89.56 nm, respectively, all below 100.0 nm. On the other hand, the mixed NEOF-4 formulation with a ratio of 2:1:1 resulted in a particle size larger than 100 nm (Table 2).

**Table 2:** Particle size, polydispersity index (PDI), and zeta-potential of various nanoemulsions of essential oils, chemical standards, and essential oil formulas at 1.0% in water.

Nanoemulsions (1.0% in water)	Particle size (nm)	PDI	Zeta potential (mV)
Plant essential oil-based nanoemulsions (NEOs)			
NEO-CL (clove:Tween60:PEG400 = 2:9:2)	20.72 ± 0.43	0.28 ± 0.01	−0.28 ± 1.53
NEO-CI (cinnamon:Tween60:PEG400 = 2:9:2)	20.80 ± 0.24	0.23 ± 0.01	−3.03 ± 0.53
NEO-TU (turmeric:Tween 80:PEG400 = 2:6:3)	18.18 ± 1.87	0.30 ± 0.01	−4.59 ± 0.37
Nanoemulsions of main compound standards (NCSs)			
NCS-E (eugenol:Tween60:PEG400 = 2:9:2)	20.44 ± 0.30	0.24 ± 0.01	−3.28 ± 0.87
NCS-T (turmerone:Tween 80:PEG400 = 2:6:3)	17.15 ± 1.76	0.32 ± 0.02	−3.43 ± 0.91
Nanoemulsion-based plant essential oil formulations (NEOFs)			
NEOF-1 (NEO-CL:NEO-CI:NEO-TU = 4:0:0)	20.76 ± 0.44	0.34 ± 0.02	−4.89 ± 0.92
NEOF-2 (NEO-CL:NEO-CI:NEO-TU = 2:2:0)	20.66 ± 0.47	0.30 ± 0.03	−3.06 ± 0.71
NEOF-3 (NEO-CL:NEO-CI:NEO-TU = 2:0:2)	89.56 ± 0.36	0.52 ± 0.02	−1.96 ± 0.65
NEOF-4 (NEO-CL:NEO-CI:NEO-TU = 2:1:1)	103.05 ± 2.68	0.27 ± 0.03	−1.52 ± 0.94

**Figure 1:**
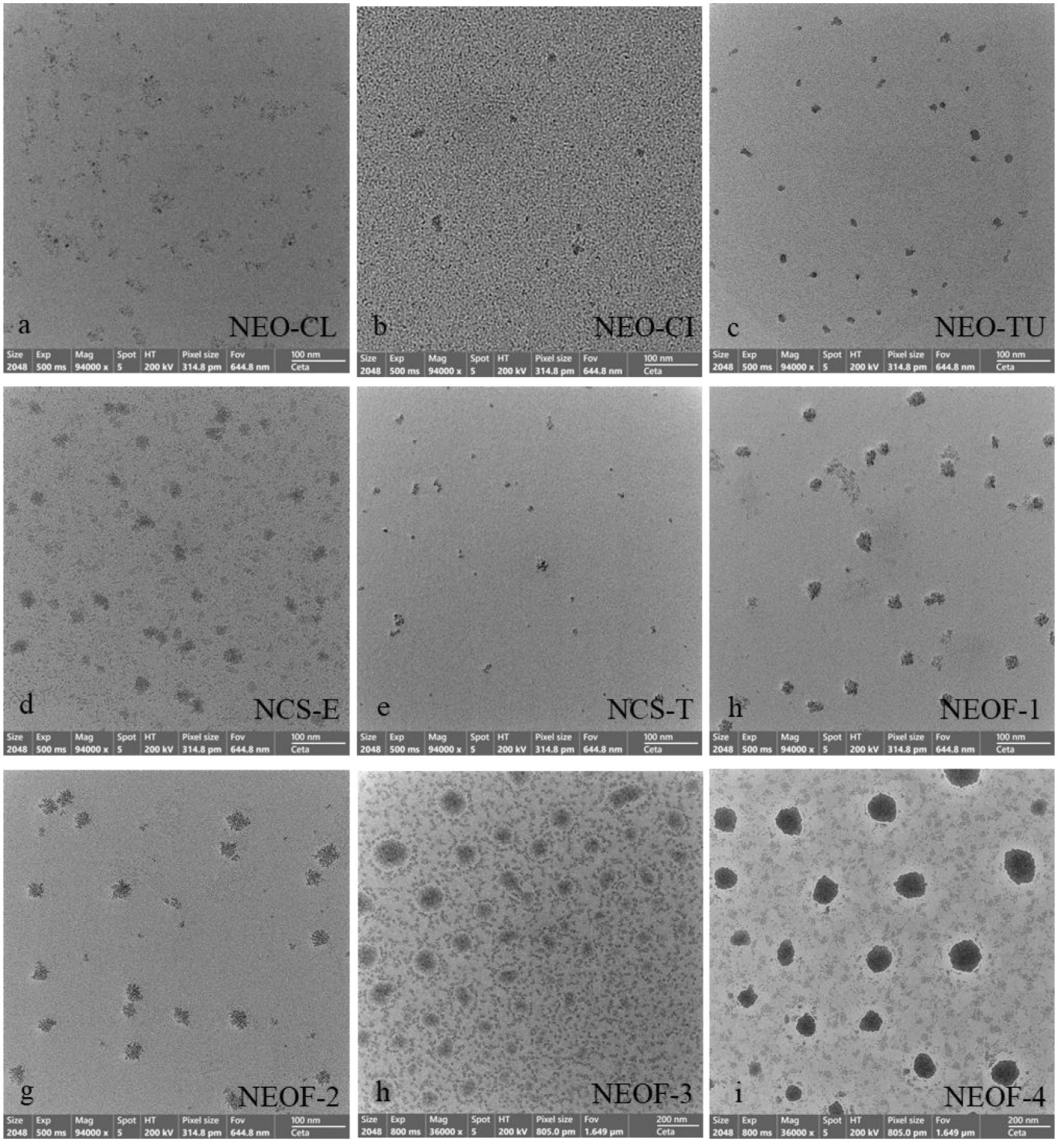
TEM morphological analysis of various nanoemulsions of essential oils, chemical standards and essential oil formulas at 1.0% in water, observed at a magnification of 94,000× (A–G) and 36,000× (H and I). NEOFs are the mixture of various NEOs with different ratio, NEOF-1 (NEO-CL:NEO-CI:NEO-TU = 4:0:0), NEOF-2 (NEO-CL:NEO-CI:NEO-TU = 2:2:0), NEO-CL (clove:Tween60:PEG400 = 2:9:2), NEO-CI (cinnamon:Tween60:PEG400 = 2:9:2), NEO-TU (turmeric:Tween 80:PEG400 = 2:6:3).

TEM images of the nanoemulsion-based plant EO formulations revealed rough-shaped droplets with opaque and nonsmooth surfaces, exhibiting particle sizes ranging from 17.15 to 20.80 nm for NEOs, NCSs, and NEOF-1 and NEOF-2 formulations. Additionally, smooth-surfaced spherical droplets were observed with particle sizes of 89.56 nm and 106.02 nm for NEOF-3 and NEOF-4 formulations, respectively (Figure 1).

### Contact Toxicity and Repellent Efficacy

**Figure 2:**
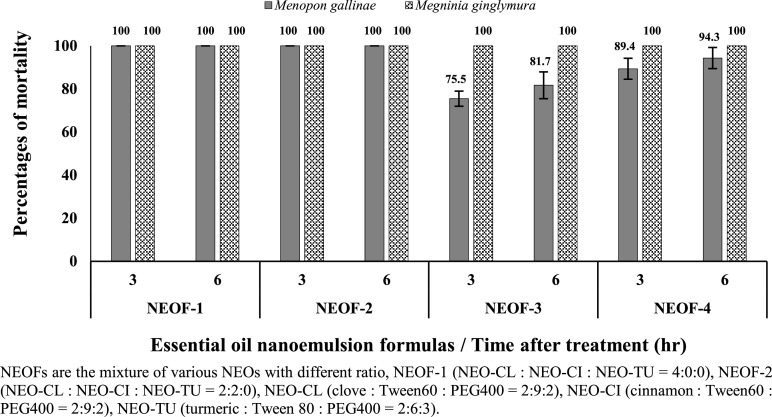
Percentages of mortality of the shaft louse (*Menopon gallinae*) and the feather mite (*Megninia ginglymura*) caused by different nanoemulsion-based plant essential oil formulations (NEOFs) at 0.3% concentration by contact method after various exposure times.

The effectiveness of the NEOFs, obtained by mixing various NEOs with different ratios, was evaluated. NEOF-1 and NEOF-2, at a concentration of 0.3%, exhibited complete toxicity against both ectoparasites (*M. gallinae* and *M. ginglymura*) at 3 h after treatment. Similarly, NEOF-3 and NEOF-4, at the same concentration, showed high efficiency in killing *M. ginglymura* (100%), but their effectiveness against *M. gallinae* was less than 90% (Figure 2). NEOF-1 and NEOF-2, identified as the most effective formulations against both ectoparasites, were selected for further toxicity testing at concentrations ranging from 0.15 to 0.35%. The results showed that these formulas achieved LC_50_ values of 0.149 to 0.160% and LC_90_ values of 0.224 to 0.242% against *M. gallinae* at 6 h after treatment. Furthermore, they exhibited toxicity levels of 0.072 to 0.120% and 0.131 to 0.204% against *M. ginglymura* at 6 h after treatment, respectively (Table 3).

**Table 3:** Toxicity values (LC_50_ and LC_90_) of different nanoemulsion-based plant essential oil formulations (NEOFs) at various concentrations against the shaft louse (*Menopon gallinae*) and the feather mite (*Megninia ginglymura*) by contact method after various exposure times.

Treatments (NEOFs)^2^	Toxicity^1^			SE	χ^2^	*P*
	Regression^3^	LC_50_ (%) (range)	LC_90_ (%) (range)			
*Menopon gallinae*						
3 h after treatment						
NEOF-1	Y = −3.639 + 19.572x	0.186 (0.166–0.202)	0.251 (0.232–0.286)	1.759	8.148	0.086*
NEOF-2	Y = −3.153 + 18.282x	0.172 (0.162–0.181)	0.243 (0.232–0.257)	1.729	1.872	0.759^ns^
6 h after treatment						
NEOF-1	Y = −2.474 + 15.507x	0.160 (0.095–0.192)	0.242 (0.208–0.331)	1.384	22.331	<0.001^⁎⁎^
NEOF-2	Y = −2.531 + 17.010x	0.149 (0.116–0.169)	0.224 (0.203–0.261)	1.638	8.589	<0.001^⁎⁎^
*Megninia ginglymura*						
3 h after treatment						
NEOF-1	Y = −2.136 + 25.049x	0.085 (0.068–0.103)	0.136 (0.117–0.173)	1.575	17.837	0.001^⁎⁎^
NEOF-2	Y = −1.990 + 14.480x	0.137 (0.116–0.176)	0.226 (0.184–0.327)	1.360	11.161	0.025*
6 h after treatment						
NEOF-1	Y = −1.548 + 21.539x	0.072 (0.048–0.095)	0.131 (0.106–0.188)	1.391	25.564	<0.001^⁎⁎^
NEOF-2	Y = −1.818 + 15.166x	0.120 (0.091–0.173)	0.204 (0.159–0.395)	1.490	12.782	<0.005^⁎⁎^

1 Data were determined based on *n* = 10 adults of ectoparasites/3 replications lethal concentrations of nanoemulsions of plant essential oil formulas (NEOFs) needed to kill 50 and 90% of the insects or mite (LC_50_ and LC_90_, respectively) at 3 and 6 h after treatment.

2 NEOFs are the mixture of various NEOs with different ratios, NEOF-1 (NEO-CL:NEO-CI:NEO-TU = 4:0:0), NEOF-2 (NEO-CL:NEO-CI:NEO-TU = 2:2:0), NEO-CL (clove:Tween60:PEG400 = 2:9:2), NEO-CI (cinnamon:Tween60:PEG400 = 2:9:2), NEO-TU (turmeric:Tween 80:PEG400 = 2:6:3).

3 Probit (Y) = Intercept + Slope × (Concentration: x), *, **: Significant difference at *P* < 0.05 and *P* < 0.01, respectively, ns: nonsignificant difference. SE: standard error, χ^2^: chi-square value.

The response effects of NEOFs, specifically NEOF-1 and NEOF-2, were examined in vitro to assess their repellent and attractant properties against the ectoparasites *M. gallinae* and *M. ginglymura*. The results revealed that the NEOFs exhibited higher repellent responses toward *M. gallinae* compared to *M. ginglymura*. At a concentration of 0.25%, the NEOFs demonstrated repellency rates exceeding 80.0% for *M. gallinae*, with a significant difference observed. Interestingly, NEOF-2 displayed higher repellent percentages against *M. gallinae* compared to NEOF-1. NEOF-1 at a concentration of 0.25% exhibited repellency rates that were not significantly different from NEOF-2. Furthermore, the repellent response of NEOFs at concentrations of 0.15 and 0.25% against *M. ginglymura* ranged from 60.0 to 73.3%, which did not significantly differ from the attractant response rates of 26.7 to 40.0%. Overall, the response effects of NEOFs across all treatments remained consistent after 3 h and 6 h of treatment (Table 4).

**Table 4:** Percentage response (repellency and attraction) of the shaft louse (*Menopon gallinae*) and the feather mites (*Megninia ginglymura*) to different nanoemulsion-based plant essential oil formulations (NEOFs) by contact method after various exposure times.

Treatments (NEOFs)^2^	Concentrations (%)	*Menopon gallinae*				*Megninia ginglymura*			
		Response^1^		χ^2^	*P*	Response^1^		χ^2^	*P*
		%R	%A			%R	%A		
3 h after treatment									
NEOF-1	0.15	63.3	36.7	1.086	0.297^ns^	70.0	30.0	2.500	0.113^ns^
	0.25	83.3	16.7	7.500	0.006^⁎⁎^	70.0	30.0	2.500	0.113^ns^
NEOF-2	0.15	83.3	16.7	7.500	0.006^⁎⁎^	60.0	40.0	0.606	0.436^ns^
	0.25	80.0	20.0	5.934	0.014*	66.7	33.3	1.714	0.190^ns^
6 h after treatment									
NEOF-1	0.15	60.0	40.0	0.606	0.436^ns^	73.3	26.7	3.455	0.063^ns^
	0.25	80.0	20.0	5.934	0.014*	66.7	33.3	1.714	0.190^ns^
NEOF-2	0.15	86.7	13.3	9.319	0.002^⁎⁎^	73.3	26.7	3.455	0.063^ns^
	0.25	86.7	13.3	9.319	0.002^⁎⁎^	73.3	26.7	3.455	0.063^ns^
Control	0.00	50	50	-	-	Control	50	50	-

1 Data were determined based on *n* = 10 adults of ectoparasites/3 replications, %R: indicates the percentage response to the treatment (repellency), %A: indicates the percentage response to the control (attraction) at 3 and 6 h after treatment, *, **: Significant difference at *P* < 0.05 and *P* < 0.01, respectively, ns: nonsignificant difference. SE: standard error, χ^2^: chi-square value.

2 NEOFs are the mixture of various NEOs with different ratios, NEOF-1 (NEO-CL:NEO-CI:NEO-TU = 4:0:0), NEOF-2 (NEO-CL:NEO-CI:NEO-TU = 2:2:0), NEO-CL (clove:Tween60:PEG400 = 2:9:2), NEO-CI (cinnamon:Tween60:PEG400 = 2:9:2), NEO-TU (turmeric:Tween 80:PEG400 = 2:6:3).

The NEOFs demonstrated high effectiveness in terms of their toxicity properties and repellent efficiency against both poultry ectoparasites. At a concentration of 0.25%, the NEOFs achieved complete mortality of both ectoparasites at 6 h after treatment, with no significant difference compared to the cypermethrin insecticide and NCS-E group. However, the NEOFs exhibited lower activity with mortality rates of less than 90% against both ectoparasites at 3 h after treatment. Regarding repellent efficiency, the NEOFs and NCS-E at a concentration of 0.25% showed a higher repellent response effect against *M. gallinae* (>80%) compared to *M. ginglymura* (>66.7%). However, their repellency rates were lower than those observed in the cypermethrin insecticide group (100%) (Table 5).

**Table 5:** Percentage of mortalities of the shaft louse (*Menopon gallinae*) and the feather mite (*Megninia ginglymura*) and repellent response of different nanoemulsion-based plant essential oil formulations (NEOFs) compared with the insecticide, NCS-E, and control groups.

Treatments^1^	After treatments (h)	Toxicity^2^			
		*Menopon gallinae*		*Megninia ginglymura*	
		%M	%R	%M	%R
Control (surfactant 0.25%)	3	0.0	-	0.0	-
	6	0.0	-	0.0	-
Cypermethrin insecticide (0.1%)	3	100.0	100.0^a^	100.0	100.0^a^
	6	100.0	100.0^a^	100.0	100.0^a^
NCS-E (0.25%)	3	90.3	80.0^b^	100.0	70.0^b^
	6	96.7	83.3^b^	100.0	73.3^b^
NEOF-1 (0.25%)	3	93.3	83.3^b^	100.0	70.0^b^
	6	100.0	80.0^b^	100.0	66.7^b^
NEOF-2 (0.25%)	3	96.7	80.0^b^	100.0	66.7^b^
	6	100.0	86.7^b^	100.0	73.3^b^
Sig.^3^		ns	*	ns	**

1 Control (Tween60:PEG400 = 9:2), cypermethrin insecticide 35%EC (recommended dose; 0.1%), NCS-E (eugenol:Tween60:PEG400 = 2:9:2), NEOF-1 (NEO-CL:NEO-CI:NEO-TU = 4:0:0), NEOF-2 (NEO-CL:NEO-CI:NEO-TU = 2:2:0), NEO-CL (clove:Tween60:PEG400 = 2:9:2), NEO-CI (cinnamon:Tween60:PEG400 = 2:9:2), NEO-TU (turmeric:Tween 80:PEG400 = 2:6:3).

2 Data were determined based on *n* = 10 adults of ectoparasites/3 replications, %M: percentages of actual mortality, %R: indicates the percentage response to the treatment (repellency).

3 *, **: Significant difference at *P* < 0.05 and *P* < 0.01, respectively, ns: nonsignificant difference.

a,b Significantly different at *p* < 0.05.

## DISCUSSION

Plant EOs are complex mixtures of secondary metabolites produced by plants for various purposes. They can contain various compounds at different concentrations, with a few major components that determine their biological properties (Bakkali et al., 2008). In this study, eugenol was identified as the major chemical compound in clove and cinnamon EOs, accounting for over 75.00% of their composition (Table 1). This finding is consistent with previous studies by Jumbo et al. (2014), Pumnuan and Insung (2016), Jumbo et al. (2018), and Shahina et al. (2022). Turmerone was found to be the major chemical compound in turmeric EO, comprising 68.46% of its composition (Table 1). This is in line with other reports that have demonstrated turmerone as a major compound in turmeric EO, accounting for around 60% of its composition, possibly in the forms of *ar*-turmerone, α-turmerone, and β-turmerone (Jayaprakasha et al., 2005; Liju et al., 2011; Jaiswal and Naik, 2021). However, variations in EO composition can occur due to factors such as geographical location, season, growth stage, and plant parts used.

Most of the emulsion treatments (NEOs, NCSs, and NEOFs, except NEOF-4) at a concentration of 1.0% in water exhibited a nanoemulsion form, with mean droplet sizes below 100 nm (Table 2). Emulsions are isotropic dispersions of 2 nonmiscible liquids, oil, and water. If the mean droplet size is between 100 and 400 nm, they are referred to as microemulsions (Cimino et al., 2021). These nanoemulsions are translucent or cloudy and are created by emulsifying the oily and aqueous phases using an emulsifier, resulting in a stable colloidal system (Perumal et al., 2021). The selection of emulsifier type and oil-to-emulsifier ratios was carefully considered. Nanoemulsions of EOs in water have been shown to enhance the bioavailability and diffusion of EOs due to the wetting ability of surfactants. Cosurfactants are often required because their free energy is higher than that of separate oil and water phases (Cimino et al., 2021). Many studies have reported the use of surfactants and cosurfactants as emulsifiers in nanoemulsions of EOs in water, including poloxamer 188, lecithin, PluronicF68, Tween60, Tween80, decanoyl/octanoyl-glycerides, glycerin monostearate, span 8, and polyethylene glycol 400 (**PEG400**) (Shi et al., 2016; Cimino et al., 2021; Doungnapa et al., 2021; Singh et al., 2021; Yahya et al., 2022). In this study, Tween60 and Tween80 were used as surfactants, and PEG400 was used as a cosurfactant in the nanoemulsion-based formulations of different oils and their ratios, following the recommendations of Doungnapa et al. (2021). The PDI values obtained in this study ranged from 0.02 to 0.34, indicating homogeneity in the particle size distribution of the nanoemulsions (Table 2). A low PDI value (<0.3) indicates a highly stable nanoemulsion, while a higher PDI value (>0.7) suggests a lower degree of uniformity and extensive particle size distribution (Perumal et al., 2021).

The zeta potential values obtained in this study ranged from −4.59 to −0.28 mV, indicating negative zeta potentials (Table 2). This can be attributed to factors such as nonionic surfactants, the negative charge of EO droplets, the adsorption of negative ions on the surface of EO droplets, and the presence of functional groups in the chemical constituents of EO (Perumal et al., 2021). Morphological analysis of the nanoemulsion-based EO formulations revealed opaque and nonsmooth surfaces for particle sizes above 20 nm, while dark and spherical surfaces were observed for nanoparticle sizes larger than 78 nm. These morphological characteristics are similar to the findings reported by Perumal et al. (2021), where nanoemulsions from EOs with diameters ranging from 70 to 100 nm exhibited a dark and spherical morphology. Kumari et al. (2018) also observed bright and smooth-surfaced spherical structures in thymol nanoemulsions ranging from 90 to 180 nm. Interestingly, nanoemulsions with nanometric micelle diameters (<20 nm) exhibited nonsmooth surfaced micellar droplets, similar to the findings reported by Moradi and Barati (2019) of rough spherical shapes.

The efficacy of the NEOFs depended on the specific chemical compounds present in the oils. NEOF-1 contained only clove EO, while NEOF-2 integrated both clove and cinnamon EOs (Figure 2). This finding aligns with previous research by Pumnuan et al. (2020), which suggested that combining clove EO with cinnamon or turmeric EOs could be used as an alternative medicinal insecticide for controlling chicken lice on farms. In this study, the combination of clove and cinnamon EOs demonstrated higher activity against the ectoparasites *M. ginglymura* and *M. gallinae* compared to the combination with turmeric EO (Figure 2). Although clove EO has been shown to have higher insecticidal activity than cinnamon EO against bean weevils (Jumbo et al., 2014), cowpea weevils (Jumbo et al., 2018), thrips, mealybugs (Pumnuan and Insung, 2016), and maize weevils (Gonzales Correa et al., 2015), its potential is lower in combination conditions. Numerous studies have demonstrated that the combination of EOs exhibits higher pesticide potential than pure EOs, as observed in antimicrobial activity (Sukatta et al., 2008; Goñi et al., 2009) and insecticidal activity (Benelli et al., 2017; Ríos et al., 2017). The NEOFs in this study exhibited higher pesticidal activity against *M. ginglymura* than *M. gallinae*, additionally, it was observed LC_50_ and LC_90_ value of *M. ginglymura* lower than *M. gallinae* (Table 3, Table 4, Table 5). This might be due to the smaller size of *M. ginglymura* (approximately 0.5 mm in length) compared to the shaft louse *M. gallinae* (approximately 2.0 mm in length). However, when applied as a pesticide to control *M. gallinae*, the NEOFs also exhibited effectiveness against *M. ginglymura*. Conversely, the repellent effect tests of NEOF-1 and NEOF-2 demonstrated higher repellent activity against *M. gallinae* than *M. ginglymura* (Tables 4 and 5), likely because *M. gallinae* is more mobility greater than *M. ginglymura*. The repellent effect of NEOF-1 and NEOF-2 might be attributed to eugenol, which serves as an active ingredient and is the major chemical compound in clove and cinnamon EOs. Also, several reports that eugenol has been shown to be an effective repellent against the other insect and mites (Ogendo et al., 2008; Al-Harbi et al., 2021; Abenaim et al., 2022; De Jorge et al., 2022).

To enhance the efficiency of botanical pesticides, the use of NEOFs is recommended, as nanoemulsions have been shown to significantly increase the stability compared to pure EO (Firooziyan et al., 2022). Future research should focus on applying NEOFs as nanoemulsion formulations based on clove and cinnamon EOs under poultry farm conditions to further explore their potential efficacy. Overall, the findings of this study contribute to our understanding of the specific chemical compounds in EOs, the formulation and stability of NEOFs, and their potential as botanical pesticides.
